# Compliance to specifications in an external quality assurance program: did new biological variation estimates of the European Federation of Laboratory Medicine (EFLM) affect the quality of laboratory results?

**DOI:** 10.1515/almed-2023-0155

**Published:** 2023-11-23

**Authors:** Carmen Ricós, Carmen Perich, Sandra Bullich, Montserrat Ventura, Berta Piqueras, Mariona Panadés, Pilar Fernández-Calle

**Affiliations:** External Quality Programs Workgroup of the Spanish Society of Laboratory Medicine (SEQC^ML^), Barcelona, Spain; Department of Laboratory Medicine, La Paz University Hospital, Madrid, Spain

**Keywords:** external quality assurance, analytical performance specification, biological variation

## Abstract

**Objectives:**

The results of external quality assurance schemes are evaluated against specifications generally based on biological variation (BV) data. This study was carried out to determine whether new BV values affected the level of compliance to specifications. Our secondary objective was to identify the conditions that would be compromised as a result of poor analytical performance in disease associated markers.

**Methods:**

This study was based on the results of the SEQC^ML^ External Quality Assurance scheme for the 2015–2022 period. Deviation of the individual result from the target value was estimated. Additionally, we calculated the percentage of results that met the pre-established specification.

**Results:**

In 97 of the 133 analytes, the level of compliance was maintained in 80–90 % of the results obtained in the two study periods. In 23 analytes, the level of compliance ranged from 51 to 79 % in the two study periods. In ALT, AST and sodium, the level of compliance was ≤50 % of the results obtained in the first study period, with sodium being the only analyte that maintained this poor level of compliance in the second study period.

**Conclusions:**

The level of compliance to specifications remained independent from the specification used (SEQC^ML^ or EFLM) for the majority of the analytes. The results for sodium ion were below the target value, which may lead to misdiagnosis of hyponatremia. Non-compensated alkaline picrate methods overestimate creatinine, which may produce false information suggestive of kidney failure.

## Introduction

External quality control (EQA) in laboratory medicine is an activity of laboratory medicine performed by an external entity involving the scheduled distribution of control samples and evaluation of the results obtained, as detailed in the ISO/IEC Guide 43 standard [1]. The purpose of EQA is monitoring analytical methods, as recognized in ISO/IEC Guide 43 [1] and European regulation IVD 2017/746 [2]. EQA places the focus on evaluating the diagnostic performance of laboratory test-analytes [3, 4] and is a requirement of the ISO 15189 standard for the accreditation of clinical laboratories [5].

SEQC^ML^ has offered 17 monthly or quarterly external quality assurance schemes (EQA) in annual cycles since 1980 [6]. A total of 919 laboratories participated in the last period evaluated (2022). The results of the participating laboratories are evaluated against a specification based on biological variation (BV), where available.

Specifications based on biological variation were described in 1999 and were modified in 2019, when the European Federation of Clinical Chemistry and Laboratory Medicine (EFLM) database was published.

The objectives of this study included:

– Investigating whether compliance with quality standards based on BV has changed as a result of the application of the new EFLM estimates. These estimates were obtained by performing a critical review and meta analysis of the EQA results obtained by the Spanish Society of Laboratory Medicine (SEQC^ML^) during an eight-year period.
– Identifying the test-analytes that do not meet specifications, thereby requiring laboratories and the *in vitro* diagnostics industry to improve their analytical performance. The resulting corrective actions would prevent misdiagnoses or erroneous monitoring results. Another goal was to identify the conditions that are most compromised by poor analytical performance.

## Materials and methods

The study was based on EQA data and is focused on nine analytical-phase EQA programs for quantitative tests.

Results were extracted from the EQA management system developed by SEQC^ML^. Data processing involved:

(1) Data extraction: Extracting individual anonymized laboratory results per month (round) and scheme.
(2) Grouping of results: provided that there are more than 10 accepted results per test-analyte, a peer group is established (same method/instrument). When methods differ (same method with different instruments) and 10 results are not available, all laboratories are included in the group.
(3) Detecting and removing outliers, i.e., values iteratively above or below the mean ±3 SDs, until all values fall within this interval [7].
(4) Estimating deviation of the individual result from the target value considered in each program, expressed as a percentage deviation; this being an estimate of total error (TE) of the analytical process.
(5) Calculating the percentage of results (percentile) meeting the specification for the TE defined in each round, cycle and program.

Two periods were considered in this study. In the first study period (2015–2018), the level of compliance was calculated against specifications based on the SEQC^ML^ BV database [8]. In the second study period, (2019–2022) specifications were based on the EFLM BV database [9]. Meeting a specification involves obtaining a total analytic error equal or lower than the specification value. The level of compliance is calculated from the highest percentile meeting the specification.

In serum-commutable-biochemistry and serum-commutable-cardiovascular risk programs, the target value is the value assigned by a certified reference method. In the other programs, the target value is the mean value obtained by laboratories in the peer group (method/instrument) or the mean value of the method or the total.

Analytical performance specifications are based on BV as they make it possible to control analytical noise. Therefore, variation in individual serial results can be explained by the sum of physiological fluctuation and analytical variation [10, 11].

## Results

Programs, analytes, evaluation criteria, and specifications were detailed in Tables 1 [Table j_almed-2023-0155_tab_002]–3. Tables are structured according to the category of BV on which the specification is based i.e., optimal, desirable or minimum [12].

**Table 1:** Analytes with specifications based on optimal BV.

Analyte	Program	Specification, %	
		SEQC	EFLM
Alpha-amylase	SUE	7.3	6.6
Alpha-amylase	SCR	7.3	6.6
Alanine aminotransferase	SBQ	13.3	8.0
Alanine aminotransferase	SCR	13.3	8.0
Aldosterone	HOR	23.3	21.4
Alpha-fetoprotein	HOR	10.9	17.4
Alpha-fetoprotein	TUM	10.9	17.4
Carcinoembryonic antigen	HOR	12.4	13.5
Carcinoembryonic antigen	TUM	12.4	13.5
Prostate-specific antigen	TUM	ND	8.1
Aspartate aminotransferase	SBQ	8.3	6.8
Aspartate aminotransferase	SCR	8.3	6.8
CA 125 antigen	TUM	17.3	8.1
CA 15-3 antigen	TUM	10.4	10.4
CA 19-9 antigen	TUM	23.0	18.9
CYFRA 21-1 antigen	TUM	13.9	13.9
Direct bilirubin	SBQ	22.3	22.3
Total bilirubin	SBQ	13.5	12.4
Total bilirubin	SCR	13.5	12.4
Cholesterol	SBQ	4.5	4.3
Cholesterol	RCV	NA	4.3
Cortisol	HOR	11.4	16.3
Creatine kinase	SUE	15.2	11.3
Creatine kinase	SCR	15.2	11.3
Estradiol	HOR	13.4	8.7
Rheumatoid factor	PRO	13.5	6.7
Folate	TUM	19.5	19.5
Folitropin	HOR	10.3	10.6
Non-esterified phosphate	SUE	11.0	5.1
Alpha1-globulin fraction, %	PRO	7.9	ND
Gamma-globulin fraction, %	PRO	8.4	ND
Gamma glutamyltransferase	SUE	11.1	9.4
Gamma glutamyltransferase	SCR	11.1	9.4
Haptoglobin	PRO	13.6	8.5
Iron (II + III)	SUE	15.3	15.3
Immunoglobulin M	PRO	8.4	8.5
Insulin	HOR	ND	15.7
Lactate	POCT	15.2	NA
Lutropin	HOR	14.0	14.2
Prolactin	HOR	14.7	11.9
C-reactive protein	PRO	28.3	25.4
Ultrasensitive C-reactive protein	RCV	NA	36.5
Thyroglobulin	TUM	11.0	14.9
Thyrotropin	HOR	11.9	13.9
Triglyceride	SUE	13.0	13.7
Triglyceride	RCV	NA	13.7
Troponin T (quantitative)	CAR	24.5	24.5
Urate	SUE	6.0	6.0
Urate	SCR	6.0	6.0
Urea	SUE	7.8	8.9

NA, not included in the EQA until 2021; ND, values not available on the database; CAR, cardiac markers; GAS, blood gases; HbA_1C_, glycosylated hemoglobin; HOR, hormones; PRO, proteins; SUE, basic non-commutable serum biochemistry; SCR, commutable serum with reference values-biochemistry; RCV, commutable serum with reference values-cardiac risk; TUM, tumor markers.

**Table 2: j_almed-2023-0155_tab_002:** Analytes with specifications based on desirable BV.

Analyte	Program	Specification, %	
		SEQC	EFLM
17-Alpha-OH progesterone	HOR	39.7	35.3
Alpha1-acid glycoprotein	PRO	16.2	12.4
Androstenedione	HOR	23.5	23.5
Free prostate specific antigen	TUM	ND	17.5
Apolipoprotein B	PRO	11.5	11.5
Apolipoprotein B	RCV	NA	11.5
HDL cholesterol	SBQ	11.3	11.1
HDL cholesterol	RCV	NE	11.5
C3 complement	PRO	8.4	7.8
C4 complement	PRO	16.0	12.1
Creatinine	SBQ	8.9	7.5
Creatinine	SCR	8.9	7.5
Creatine kinase isoenzyme, mass	CAR	16.5	ND
Neuroal specific enolase	TUM	NA	14.0
Alpha2-globulin fraction, %	PRO	12.6	ND
Beta-globulin fraction, %	PRO	11.7	ND
Ferritin	HOR	16.9	16.9
Ferritin	TUM	16.9	16.9
Alkaline phosphatase	SUE	12.0	10.6
Alkaline phosphatase	SCR	12.0	10.6
Sex hormone-binding globulin	HOR	20.4	17.2
Glucose	POCT	7.0	6.5
Glucose	SUE	7.0	6.5
Glucose	SCR	7.0	6.5
Hemoglobin A_1C_	HbA_1C_	3.0	3.1
Immunoglobulin A	PRO	13.5	9.8
Immunoglobulin G	PRO	8.0	7.3
Lactate dehydrogenase	SBQ	11.3	7.7
Lactate dehydrogenase	SCR	11.3	7.7
Magnesium	SUE	4.8	4.8
Magnesium	SCR	4.8	4.8
Myoglobin	CAR:	26.9	26.9
NT-proBNP	CAR	13.0	13.0
Parathormone	HOR	30.2	20.0
Parathormone	TUM	30.2	20.0
pCO_2_	POCT	5.7	5.7
C-peptide	HOR	20.8	20.8
Potassium	POCT	5.6	4.8
Potassium	SUE	5.6	4.8
Potassium	SCR	5.6	4.8
Proteins	SCR	3.6	3.5
Proteins	SUE	3.6	3.5
Proteins	PRO	3.6	3.5
Testosterone	HOR	21.0	17.0
Free thyroxine	HOR	8.7	9.6
Transferrin	PRO	3.8	6.8
Transthyretin (prealbumin)	PRO	14.5	14.5
Triiodothyronine (free T3)	HOR	11.3	9.3
Troponin I	CAR	27.9	27.9

NA, not included in the EQA until 2021; ND, values not available on the database; CAR, cardiac markers; GAS, blood gases; HbA_1C_, glycosylated hemoglobin; HOR, hormones; PRO, proteins; SUE, basic non-commutable serum biochemistry; SCR, commutable serum with reference values-biochemistry; RCV, commutable serum with reference values-cardiac risk; TUM, tumor markers. NA, no data. Not included in the EQA until 2022.

**Table 3:** Analytes with specifications based on minimum BV.

Analyte	Program	Specification, %	
		SEQC	EFLM
25-OH-vitamin D	HOR	NE	30.8
Albumin	PRO	6.1	5.4
Albumin	SUE	6.1	5.4
Alpha1-antitrypsin	PRO	13.8	9.3
Apolipoprotein A1	PRO	9.1	11.2
Apolipoprotein A1	RCV	NE	11.3 revisar
Beta2-microglobulin	PRO	13.5	9.7
Kappa free light chains	PRO	NE	12.0
Lambda free light chains	PRO	NE	12.7
Total kappa light chains	PRO	12.0	NE
Total lambda light chains	PRO	NE	12.9
Ionic calcium	POCT	3.1	ND
Total calcium	SUE	3.8	3.8
Total calcium	SCR	3.8	3.8
Ceruloplasmin	PRO	11.9	12.2
Chloride	POCT	2.2	2.0
Chloride	SUE	2.2	2.0
Chloride	SCR	2.2	2.0
LDL cholesterol (direct)	SUE	17.8	20.5
LDL cholesterol (direct)	RCV	ND	20.5
Homocysteine	CAR:	23.2	23.2
Lipase	SUE:	27.9^a^	21.3
Osmolality	SUE	2.3	2.3
Pseudo-cholinesterase	PRO	ND	14.7
S100	TUM	ND	25.5
Sodium	POCT	1.1	1.0
Sodium	SUE	1.1	1.0
Sodium	SCR	1.1	1.0
Dehydroepiandrosterone sulfate	HOR	19.6	15.6
Total thyroxine	HOR	10.4	13.0
Total triiodothyronine	HOR	9.2^a^	17.4

^a^Evaluated based on the desirable BV until 2018. NE, not included in the EQA until 2021; NA, values not available on the database.

The EQA programs include a total of 184 analytes. Of them, 133 are evaluated on the basis of BV data (Tables 1 [Table j_almed-2023-0155_tab_002]–3). The remaining 51 analytes were evaluated with respect to the 90th percentile (results obtained in the SEQC^ML^ scheme of the previous years) and were not included in this study.

As many as 41 % of the analytes evaluated against a specification based on BV were evaluated against the optimal specification; 36 % against the desirable specification; and 23 % against the minimum specification. The percentage of analytes evaluated against the optimal specification increased slightly in the second study period, in detriment of the desirable or minimum categories.

### Compliance to specifications based on BV

The level of compliance is quantified from the percentile of results with a deviation (with respect to the target value) equal or lower than the established specification.

The sources of the specifications used in our schemes were the SEQC^ML^ database [8] in the 2015–2018 period and the EFLM database [9] in 2019–2022.

Regardless of the source, the same category of specification (minimum, desirable or optimal) was maintained for most of the analytes in the two periods, albeit with some changes:

– In 27 analytes, desirable specifications shifted to optimal, as laboratories improved their analytical performance.

In 10 analytes, less strict specifications were used (i.e., they changed from desirable to minimum) due to the more ‘tight’ values established on the EFLM database (total triiodothyronine, parathormone, alpha1-antitrypsin, complement C4, apolipoprotein A, lactate dehydrogenase, lipase, homocysteine). Some specifications changed from the optimal to the desirable category, as laboratories failed to meet the optimal category in analytes for which only data from the SEQC^ML^ database was available (CK-MB and C peptide).

Considering all the analytes included, the overall level of compliance was virtually the same in the two study periods (83 and 84 %, respectively). Supplementary Figures 1–7 shows the level of compliance of each analyte per EQA scheme.

In relation to the evolution of the level of compliance, the 133 analytes were divided into three groups:

- In 95 of the 133 analytes, the level of compliance remained between 80 and 100 % of the results obtained, in the two study periods. This finding suggests good compliance.
- In 23 analytes, the level of compliance ranged from 51 to 79 % in the two study periods. In 12 analytes, data was not available in the first study period. This means an intermediate level of compliance.
- In alanine aminotransferase (ALT), aspartate aminotransferase (AST) (SCR scheme) and sodium (SCR and SUE schemes), the level of compliance was ≤50 % of the results in the first period, with sodium being the only analyte that showed the same level of compliance in the second period. This means an insufficient level of compliance.

**Figure 1:**
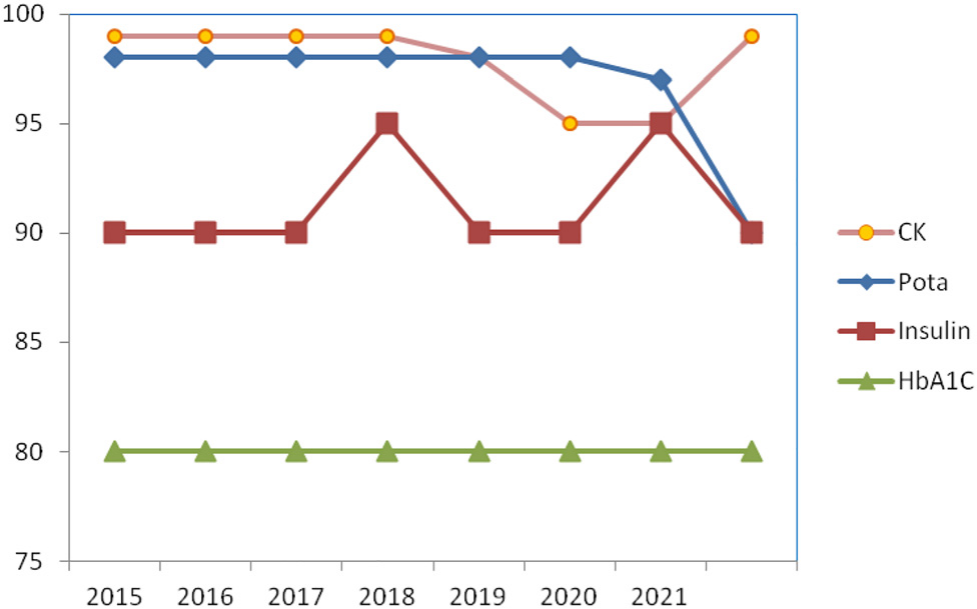
Evolution in the level of compliance to specifications. Example of analytes with good performance. CK, creatine kinase; Pota, potassium.

Figure 1 shows examples of analytes with good analytical performance included: potassium in the serum program, which always exceeded 95 % of results meeting specifications; creatine kinase (CK) and insulin, which results met between 80 and 90 % of specifications every year, as well as HbA_1c_, which maintains compliance in 80 % of the data provided to the programs.

**Figure 2:**
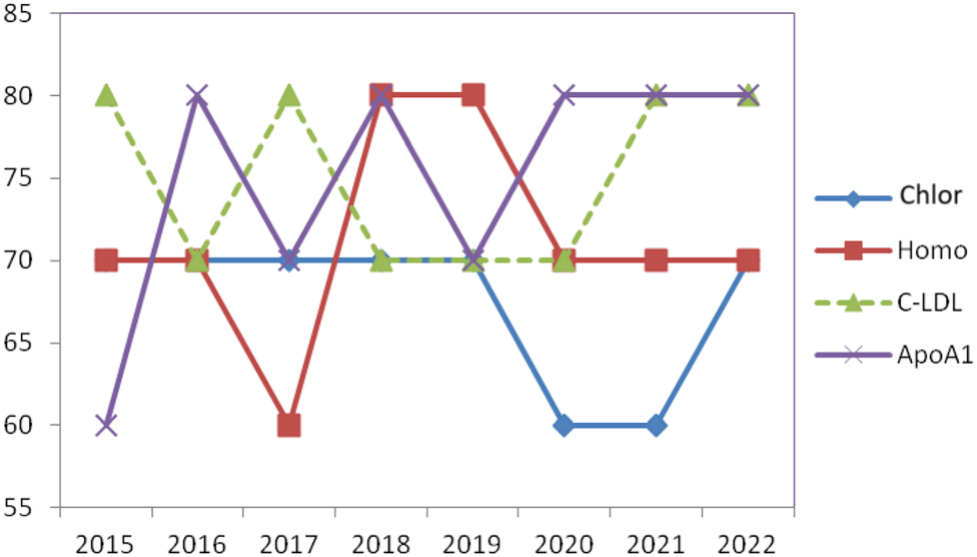
Evolution in the level of compliance to specifications. Example of analytes with intermediate performance. Chlor, chloride; Homo, homocysteine; LDL-C, LDL cholesterol; ApoA1, apolipoprotein A1.

Figure 2 summarizes some of the analytes with an intermediate performance.

**Figure 3:**
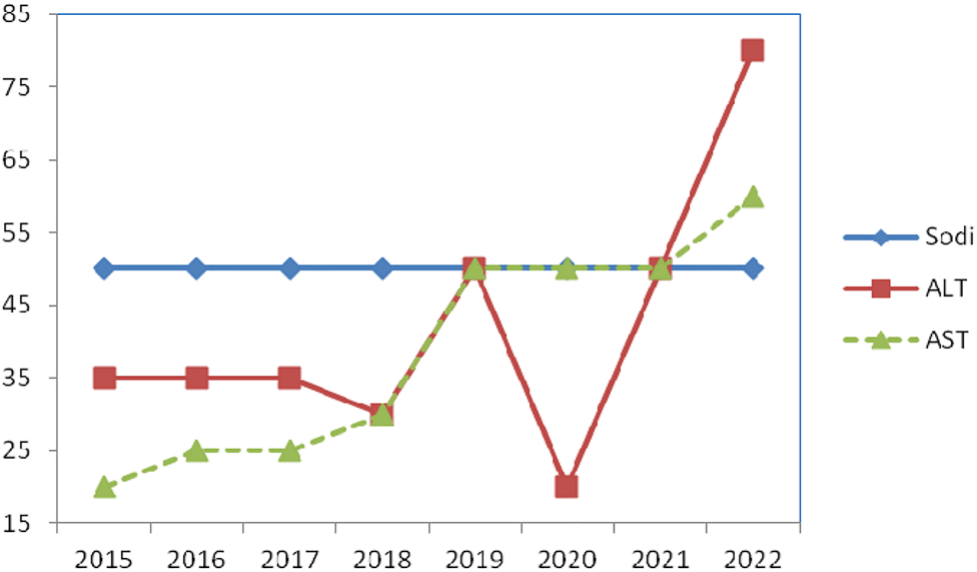
Evolution in the level of compliance to specifications. Analytes with poor performance. Sodi, sodium; ALT, alanine aminotransferase; AST, aspartate aminotransferase.

Figure 3 shows the analytes with insufficient performance included sodium (serum program); ALT and AST (SCR scheme).

## Discussion

The EQA schemes aimed at promoting improvements in laboratory performance (as SEQC^ML^ EQA schemes) should use strict quality specifications. This approach will encourage laboratories to use of internal quality control protocols to detect and prevent errors affecting information about the health status of the patient. In contrast, mandatory EQA, aimed at preventing inacceptable performance, establish broader limits of acceptability. The purpose is that only a small number of inefficient laboratories are excluded. An example is the 1 and 2 % of laboratories that are excluded by CLIA [13].

In the international scene, the use of EQA schemes based on BV increased from 19 % in 1996 to 42 % in 2017 [6], [7], [8], [9], [10], [11], [12], [13], [14]. Indeed, the USA Clinical Laboratory Improvement Amendments (CLIA), a proficiency testing program, established its specifications for 2019 on the basis of BV data [15, 16].

The present study evaluated the analytical performance of laboratories participating in SEQC^ML^ EQA schemes during the 2015–2022 period. This period includes the two BV databases: the first four years were evaluated according to the latest update of the SEQC^ML^ database [8]. The last four years were evaluated with respect to the revised estimates on the EFLM database [9].

EFLM specifications are slightly stricter than SEQC^ML^ ones. For this reason, there is some criticism that EFLM specifications have led laboratories to use the minimum category and reduce their level of analytical performance [17]. However, the results of this study contradict this opinion. In the present study, the level of compliance remained constant for most of the analytes included, regardless of the level of stringency of the specifications used to evaluate laboratory performance. Indeed, although some laboratories changed their specification category (from minimum to desirable, and from desirable to optimal in some cases), the level of overall compliance remained the same.

### Evolution of the level of compliance to specifications

In our study, compliance was considered good when 80 % or more results met specifications. Compliance is intermediate when 50–79 % of results meet specifications. Compliance is insufficient when less than 50 % of results meet specifications. These criteria are consistent with those used by Panteghini et al. [18], who defined specifications on the basis of the state of the art. The authors deemed it appropriate to use these stratification criteria, even though they are applied to different data.

Performance has improved over time for some analytes. This improvement may be due to the promotion of specifications based on BV in EQA schemes [19]. Thus, as stated above, the overall percentage of compliance was 84 %. In general, analyte quantifications in the laboratories participating in SEQC^ML^ EQA schemes showed an excellent quality.

SEQC^ML^ offers two category-1 schemes (maximum level) with replicated analyses of commutable controls and values assigned by certified reference methods [20, 21]. These two schemes are the commutable serum with reference values program (SCR) and the cardiovascular risk program (RCV), where the level of standardization of analytical performance is evaluated.

The analytes included in the SCR program showed an acceptable level of compliance to specifications (77 %). However, this percentage is slightly lower than the one obtained in the basic non-commutable serum biochemistry program (SUE) (85 %) for common analytes. Differences in analytical performance across peer groups are adequately harmonized (non-commutable serum program). However, some groups have difficulty reaching the certified reference value (SCR program). The most relevant examples include:

– AST and ALT, due to the high number of laboratories that do not include pyridoxal-phosphate in the reagent, thereby obtaining results considerably below the reference value [14, 22].
– Calcium, with inacceptable results below the reference value in the 2015–2017 period, and with satisfactory results in the 2018–2021 period. The reason is the less frequent use of a calibrator traceable to an aqueous reference material in favor of a serum-based calibrator, as recommended by Panteghini and Ambruster [23, 24].
– Protein, for which the level of compliance to specifications has decreased over time (from 80–90 to 65 %). This could be due to the fact that an instrument used by many participants (Roche c701 and c702) yielded satisfactory results until 2019. However, it showed a negative deviation in 2020 and 2021, most frequently at concentrations >80 g/L, without any apparent change in the reagent (Biuret) or in analytical traceability of the calibrator (NIST-SRM 927). This finding demonstrates that it is necessary to promote collaboration with the manufacturer to investigate potential changes in the chain of traceability, from the master-lot to the routine calibrator. Greg Miller et al. supported this opinion in a recent paper [25]. This problem has not been observed in the SUE program, as the target value is the mean value obtained in the peer group; when results for such peer group differ significantly from the true value of the analyte, the real analytical performance of the laboratory differs from the one shown by EQA results.

In the RCV program, the level of compliance ranges from 84 to 95 % of results, according to the analyte, which demonstrates good compliance.

The fact that compliance with SCR program specifications was satisfactory is relevant, as it corresponds to determinations in commutable human serum material, which faithfully reflects what happens when patient samples are analyzed.

There are some testing methods with a poor analytical performance that hinders the use of some analytes in the clinic. Thus, the diagnosis and monitoring of some conditions could be compromised by the poor analytical performance of the laboratory methods currently used, including:

– Hyponatremia
– Hypocalcemia
– Evaluation of the nutritional status – malnutrition
– Kidney failure

### Hyponatremia

Diagnosis of hyponatremia is based on the measurement of sodium ion. The specification is based on the minimum BV (TE=1.0 %).

In the SCR program, virtually all participants use indirect potentiometry. In the first study period (2015–2018), compliance was <50 %. From 2019, the level of compliance increased to 60–70 %. This may be due to the increase in the use of the group with the best performance (Roche c701 and c702) over the years. In contrast, all results were below the certified reference value (up to −2%) in all cycles, which may lead to misdiagnosis of hyponatremia.

In the SUE program, which involved 890 participants, and primarily using indirect potentiometry, only 50 % reached the specification in all cycles.

In the POCT-blood gas test program (750 laboratories), which uses direct potentiometry, dispersion across group was small (with inter-laboratory CV ranging from 0.3 to 0.9 %, depending on the instrument) in all cycles. Therefore, results were similar across groups. However, results cannot be compared against a reference material-assigned value.

Laboratories that analyze patient samples indistinctly by direct or indirect potentiometry should ensure the commutability of results and be aware of the tendency to falsely inform hyponatremia.

The *in vitro* diagnostics industry should correct the possible negative inaccuracy of its products observed in this study.

### Hypocalcemia

The analytes of interest are total calcium and ionized calcium. For total calcium, the specification is established based on the minimum BV (TE=3.8 %).

As mentioned above, the SCR program showed satisfactory results for the 2018–2021 period. The most widely used methods are the BAPTA calibrator traceable to NIST-SRM 956 (cobas series c500 and c700) and Arsenazo, with the same traceability (Architect series c).

In the measurement of total calcium, in the few laboratories that still use a, aqueous reference standard, results are consistently 10 % lower than the certified reference value. This group could falsely provide results consistent with hypocalcemia, thereby leading to unnecessary supplementation and iatrogenia.

For ionized calcium, the specification is based on the minimum BV (TE=3.1 %). This analyte is included in the POCT program. The most widespread method among participants is direct potentiometry. The target value is provided by the method/instrument group and the relative deviation of results remains constant across instruments. This means that, provided that a laboratory does not change its analytical instrument, results are consistent over time, although there is not a certified reference value for the distributed control samples.

### Evaluation of the nutritional status – malnutrition

The most widely used marker for a preliminary evaluation of nutritional status is albumin, and its specification is based on the minimum BV (TE=5.4 %).

This analyte is not included in the SCR program. Therefore, there is no reference material-assigned value available.

In the SUE program, the level of compliance remains constant at 80 %. This level of harmonization is due to the fact that most laboratories use the bromocresol green method, with very similar results. In contrast, users of the bromocresol purple method (20 % of laboratories) obtained 60 % lower results.

In the protein program (PRO), the level of compliance for albumin was 77 %. Most laboratories use the bromocresol green method, and the target value is defined by the same instrument as in the SUE program. Strikingly, the results obtained by the few laboratories that use the bromocresol purple method are slightly lower (−6%) than the ones obtained by the bromocresol green method, but not as low as in the SUE program. This difference could be due to the control material matrix, which is bovine in the SUE program and human in the PRO program.

Laboratories using the bromocresol purple method could falsely inform malnutrition, if their population-based reference values are not adjusted to their method. In addition, clinical laboratories using the two methods, either alternatively or simultaneously (emergency care/routine care), may provide confusing information.

### Evaluation of kidney function – chronic kidney disease

The analyte to study kidney function is serum creatinine, from which glomerular filtration rate (GF) is estimated using different formulas, being the CKD-EPI formula the most widespread [26]. The different formulas used, with different diagnostic sensitivity, contribute to variability in patient classification.

The specification for serum creatinine is based on the desirable BV (TE=7.5 %). The level of compliance was 80 % approximately during the 8 years of the study. Enzymatic methods (traceable to NIST-SRM 914a and 967a) and the compensated alkaline picrate method (traceable to IDMS) yield correct results. However, the non-compensated alkaline picrate method yields results >30 % higher at concentrations of 50–75 μmol/L. Unfortunately, half of participants in SEQC^ML^ programs use this method, which results in serum creatinine overestimation. This overestimation translates into poor GF estimates that classify CKD in a more advanced stage than real. This misclassification results in unnecessary follow-up, complementary studies and monitoring. Creatinine overestimation has been observed both, in the SCR and in the SUE program. This means that a high number of laboratories in Spain still use methods that overestimate creatinine to clinically relevant levels. Thus, laboratories provide false information suggestive of kidney failure, which involves unnecessary medical costs.

## Conclusions

(1) Compliance to specifications keeps constant in most analytes, regardless of how strict specifications are (SEQC^ML^ or EFLM). Indeed, although some laboratories changed of specification category (from minimal to desirable, and from desirable to optimal in some cases), the overall level of compliance remained the same.
(2) Performance has improved for some analytes over time, with a final overall level of compliance of 84 %. Therefore, quantitative determinations in laboratories participating in SEQC^ML^ EQA programs show a good level of compliance to specifications.
(3) The results for sodium ion were below the certified reference value, which may result in misdiagnoses of hyponatremia. The *in vitro* diagnostics industry should correct the possible negative bias of its products observed in this study.
(4) In the measurement of total calcium, in the few laboratories that still use a routine calibrator that is traceable to an aqueous reference standard, results are consistently 10 % lower than the certified reference value, which may produce false results suggestive of hypocalcemia.
(5) Non-compensated alkaline picrate methods overestimate creatinine, which may produce false information suggestive of kidney failure.

## Supplementary Material

Supplementary Material
