# Supplementary material for: Compliance to specifications in an external quality assurance program: did new biological variation estimates of the European Federation of Laboratory Medicine (EFLM) affect the quality of laboratory results?
Source: Adv Lab Med. 2023 Nov 23;4(4):379–86. doi: 10.1515/almed-2023-0155 (PMC10724879; doi:10.1515/almed-2023-0155)
Supplement: Supplementary file 1 — Supplementary Material [file j_almed-2023-0155_suppl_001.doc]

Complementary Figure 1. Level of compliance of analytes of the Cardiac Marker program


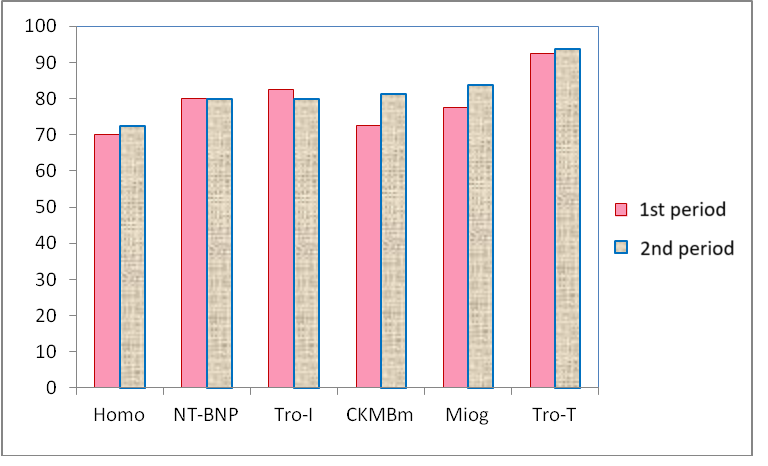


Complementary Figure 2. Level of compliance of analytes of the POCT Blood Gas Test program.


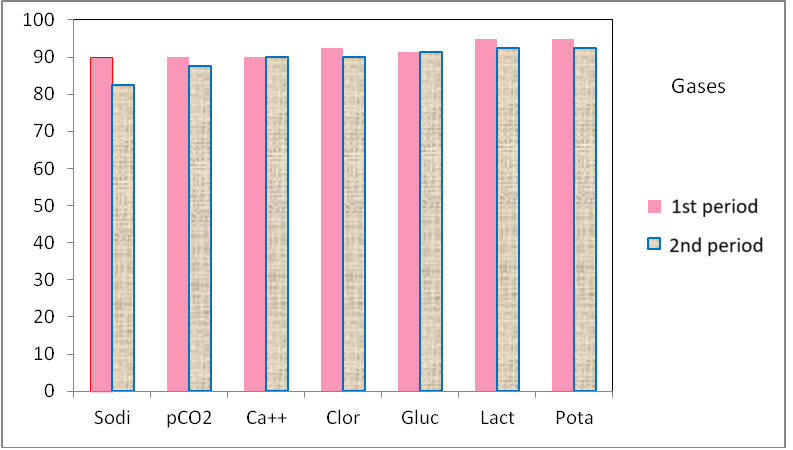


Complementary Figure 3. Level of compliance of analytes of the Hormone program


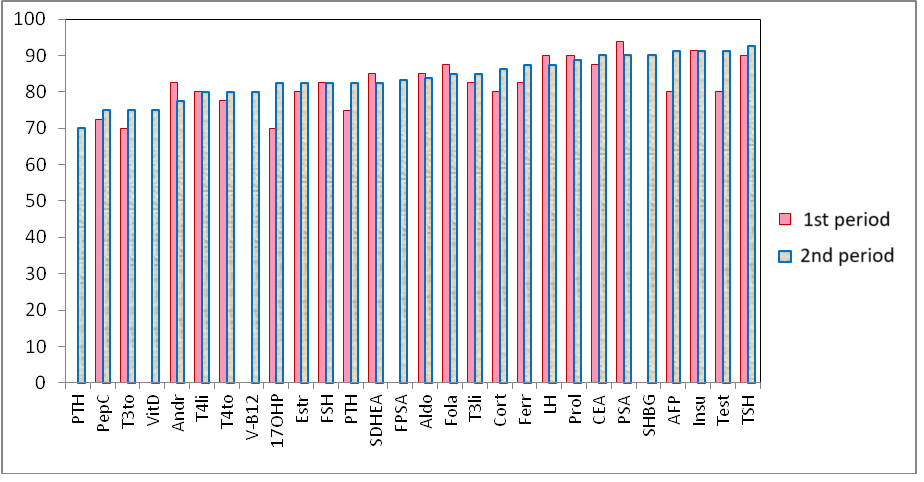


Complementary Figure 4. Level of compliance of analytes of the Protein program


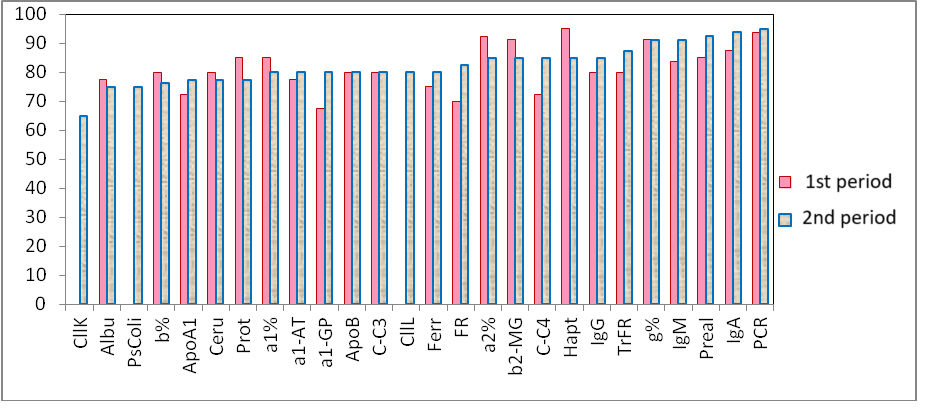


Complementary Figure 5. Level of compliance of analytes of the Tumor Marker program


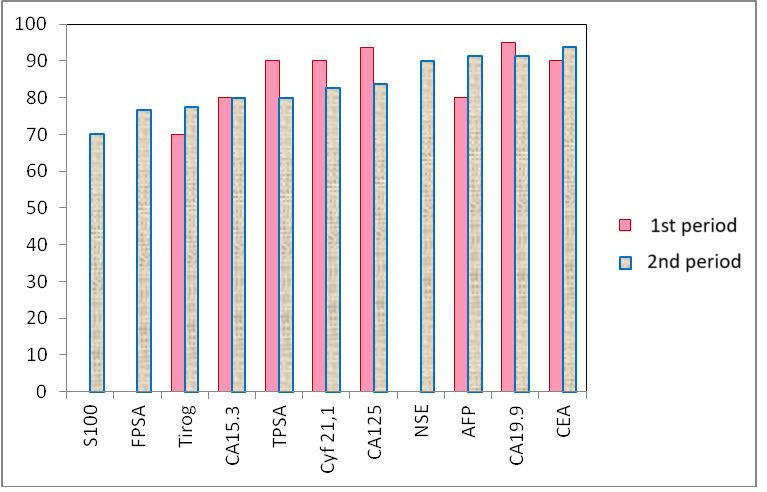


Complementary Figure 6. Level of compliance of analytes of the Basic Non-Commutable Serum Biochemistry program


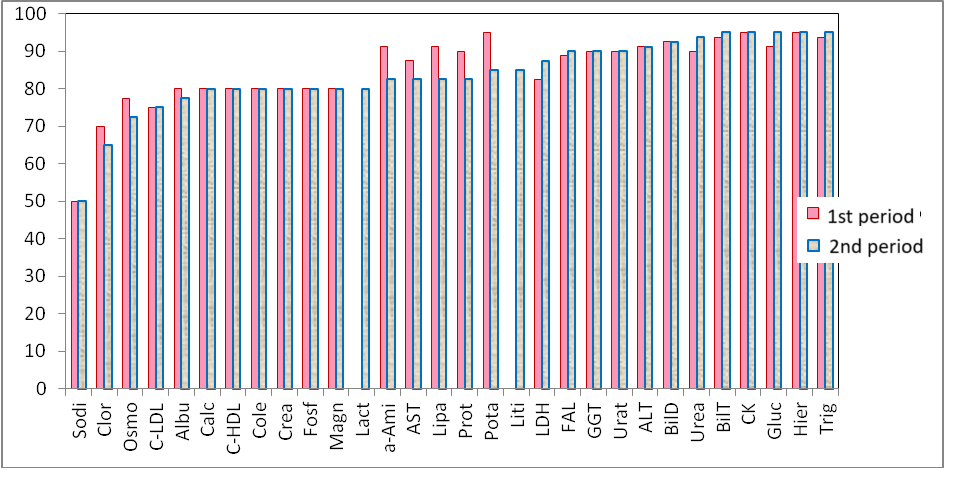


Complementary Figure 7. Level of compliance of analytes of the Commutable Serum Biochemistry program


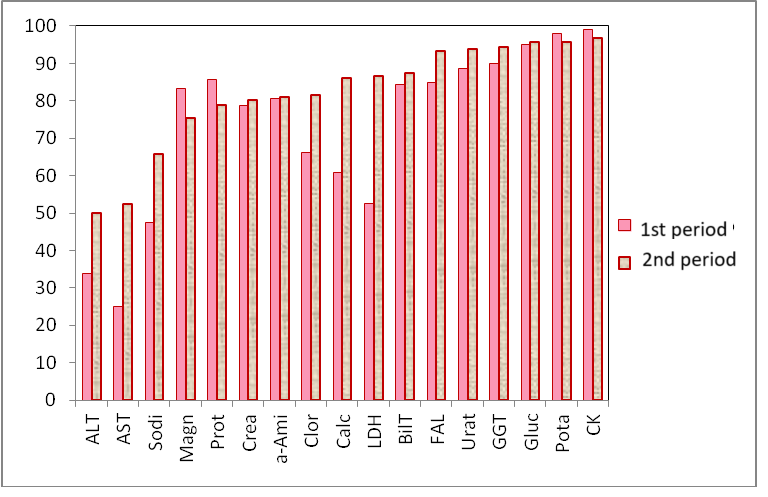


**Legend of complementary figures 1-7**

Analytes in ascending order of compliance in the second study period.

**Legend of complementary figure 1**

Homo: homocysteine; NT-BNP: natriuretic peptides; Tro-I: Troponin I: CKMBm: creatine kinase MB-mass isoenzyme; Myog: myoglobin; Tro-T: Troponin I

**Legend of complementary figure 2**

Sodi: sodium; pCO_2_: partial CO_2_pressure; Ca++: ionic calcium; Chlor: chloride: Gluc:glucosa; Lact: lactate; Pota: potasium

**Legend of complementary figure 3**

PTH: parathormone; PepC: C peptide; T3to: total triiodothyronine; VitD: 25 OH vitamin D; Andr: androstenedione; T4l: free thyroxine; T4to: total thyroxine; V-B12: vitamin B12; 17OHP: 17-α-OH progesterone; Est: estradiol; FSH: follitropin; PTH: parathomone; SDHEA: dehydroepiandrosterone sulfate; FPSA: free prostate specific antigen; Aldo: aldosterone; Fola: folate; T3li:free triiodothyronine; Cort: cortisol; Ferr: ferritin; LH: lutropin; Prol: prolactin; CEA: carcinoembryonic antigen; PSA: prostate specific antigen; SHBG: sex hormone binding globulin; AFP: alpha-fetoprotein; Insu: insulin; Test: testosterone; TSH: thyrotropin.

**Legend of complementary figure 4**

CllK: kappa free light chains; Albu: albumin; PsColi: pseudocholinesterse; b%: betaglobulin% fraction; ApoA1: apolipoprotein A1; Ceru: ceruloplasmin; Prot: protein; a1%: alphaglobulin% fraction; a1-AT: α1-antitrypsin; a1-GP: α1-acid glycoprotein; ApoB: apolipoprotein B; C-C3: complement C3; CllL: lambda free light chains; Ferr: ferritin; RF: rheumatoid factor; a2%: alpha2 globulin % fraction; b2-MG: beta2-microglobulin; C-C4: complement C4; Hapt: haptoglobin; IgG: immunoglobulin G; TrFR: transferrin; g%: gamma globulin% fraction; IgM: immunoglobulin M; Prealb: prealbumin; IgA: immunoglobulin A; CRP: C-reactive protein.

**Legend of complementary figure 5**

S100:Proteins S100; FPSA: free prostate specific antigen; Tirog: thyroglobulin; CA15.3: CA15.3 antigen; TPSA: total prostate specific antigen; Cyf21.1: cyfra 21.1 antigen; CA125: CA 125 antigen; NSE: neuronal specific enolase; AFP: alpha-fetoprotein; CA19.9: CA 19.9+ antigen; CEA: carcinoembryonic antigen.

**Legend of complementary figure 6**

Sodi: sodium; Chlor; chloride; Osmo: osmolality; LDL-C: LDL cholesterol; Chol: cholesterol; Crea: creatinine; Phosph: non-esterified phosphate; Magn: magnesium; Lact: lactate; a-Amy: alpha-amylase; AST: aspartate aminotransferase; Lipa: lipase; Prot: protein; Liti: lithium, LDH: lactate dehydrogenase; ALP: alkaline phosphatase; GGT: gamma-glutamyl transferase; Urat: urate; ALT: alanine aminotransferase; BilD: direct bilirubin; Urea: urea; BilT: total bilirubin; CK: creatine kinase; Gluc: glucose; Hier: iron (II+III); Trig: triglyceride.

**Legend of complementary figure 7**

ALT: alanine aminotransferase; AST: aspartate aminotransferase; Sodi: sodium; Magn: magnesium; Prot: protein; Crea: creatinine; a-Amil: alpha-amylase; Chlorine: chloride: Calc; calcium; LDH: lactate dehydrogenase; BilT: total bilirubin; ALP: alkaline phosphatase; Urat: urate; GGT: gamma-glutamyl transferase; Gluc: glucose; Pota: potassium; CK: creatine kinase.
